# A Nanoparticle-Based Anticaries Vaccine Enhances the Persistent Immune Response To Inhibit Streptococcus mutans and Prevent Caries

**DOI:** 10.1128/spectrum.04328-22

**Published:** 2023-03-28

**Authors:** You-Bo Yu, Ying Liu, Hangeri Liang, Xianxin Dong, Xiao-Yan Yang, Sha Li, Zhong Guo

**Affiliations:** a Center for Biological Science and Technology, Advanced Institute of Natural Sciences, Beijing Normal University, Zhuhai, Guangdong, China; b Zhuhai Key Laboratory of Basic and Applied Research in Chinese Medicine, College of Bioengineering, Zhuhai Campus of Zunyi Medical University, Zhuhai, Guangdong, China; c Instrumentation and Service Center for Science and Technology, Beijing Normal University, Zhuhai, Guangdong, China; University of Guelph College of Biological Science

**Keywords:** zeolitic imidazolate framework-8 nanoparticles, PAc, vaccine adjuvant, caries, immune response

## Abstract

Caries vaccines have been identified as a good strategy for the prevention of caries through the mechanism of inoculation against Streptococcus mutans, which is the main etiological bacterium causing caries. Protein antigen c (PAc) of S. mutans has been administered as an anticaries vaccine but shows relatively weak immunogenicity to elicit a low-level immune response. Here, we report a zeolitic imidazolate framework-8 nanoparticle (ZIF-8 NP)-based adjuvant with good biocompatibility, pH responsiveness, and high loading performance for PAc that was used as an anticaries vaccine. In this study, we prepared a ZIF-8@PAc anticaries vaccine and investigated the immune responses and anticaries efficacy induced by this vaccine *in vitro* and *in vivo*. ZIF-8 NPs substantially improved the internalization of PAc in lysosomes for further processing and presentation to T lymphocytes. In addition, significantly higher IgG antibody titers, cytokine levels, splenocyte proliferation indices, and percentages of mature dendritic cells (DCs) and central memory T cells were detected in mice subcutaneously immunized with ZIF-8@PAc than in mice subcutaneously immunized with PAc alone. Finally, rats were immunized with ZIF-8@PAc, and ZIF-8@PAc elicited a strong immune response to inhibit colonization by S. mutans and improve prophylactic efficacy against caries. Based on the results, ZIF-8 NPs are promising as an adjuvant for anticaries vaccine development.

**IMPORTANCE** Streptococcus mutans is the main etiologic bacterium of dental caries, whose protein antigen c (PAc) has been administered as an anticaries vaccine. However, the immunogenicity of PAc is relatively weak. To improve the immunogenicity of PAc, ZIF-8 NP was used as an adjuvant, and the immune responses and protective effect elicited by ZIF-8@PAc anticaries vaccine were evaluated *in vitro* and *in vivo*. The findings will help in prevention of dental caries and provide new insight for the development of anticaries vaccine in the future.

## INTRODUCTION

Dental caries is the most prevalent oral disease globally and imposes serious health and economic burdens. In 2015, a survey found that 2.4 billion people had untreated caries, affecting 34% of adults and 8% of children (1). Although various strategies have been implemented to control dental caries, the incidence of caries has remained consistently high over the past decades (2, 3). Currently, caries vaccines have been developed and are known as a good strategy for the prevention of dental caries due to the mechanism of inoculating against bacteria, particularly Streptococcus mutans, which is considered the major pathogen causing dental caries (4).

S. mutans is one of the major etiologic agents of caries. Caries vaccines can target different pathophysiological phases of S. mutans for the immunological prevention of dental caries. Over the past few decades, a number of surface or secreted products of S. mutans have been proposed as candidate antigens for caries vaccines, and attention has been focused on three protein antigens: adhesins known as protein antigen c (PAc), glucan-binding proteins, and glucosyltransferases (5). PAc, a surface fibrillar adhesin of S. mutans, is a key virulence factor associated with the initial adherence of S. mutans to tooth surfaces, which contains two main domains associated with an alanine-rich tandem repeating region (A-region) and a proline-rich repeat region (P-region) (6). Recombinant PAc is constructed with the A-region and P-region originating from the PAc protein linked with several amino acids and is utilized more commonly as an immunogen to develop a caries vaccine. This caries vaccine induces specific humoral immunity to inhibit the adherence and colonization of S. mutans on teeth (7). However, PAc shows low immunogenicity and induces low immune responses that do not maintain adequate protection against dental caries without adjuvants (6, 8).

Adjuvants, which improve antigen immunogenicity and accelerate, prolong, or enhance antigen-specific immune responses (9), are therefore required to assist PAc in eliciting sufficiently effective immune responses for protection against caries. Several adjuvants have been developed to prepare caries vaccines. Cholera toxin or heat-labile enterotoxins from Escherichia coli have been shown to be powerful adjuvants that have been applied with antigens to interrupt the proliferation of S. mutans (10, 11). However, toxin-related adjuvants may exert side effects after vaccine administration. Therefore, nontoxic derivatives or other bacterial components, which are commonly ligands of Toll-like receptors (TLRs), have been investigated in caries vaccines. FimH, an adhesin component of fimbriae from Salmonella enterica serovar Typhimurium, has been suggested and proven to be a promising adjuvant to promote immune responses via the TLR4-dependent signaling pathway and protect against caries (12). In addition, the flagellin protein derived from E. coli, a TLR5 agonist, has been specifically evaluated as an effective mucosal adjuvant for eliciting obvious mucosal and systemic immune responses (13). Furthermore, the same researchers constructed a second-generation flagellin-PAc fusion protein that elicited a robust PAc-specific IgA response and exhibited therapeutic efficiency against caries while causing fewer side effects and fewer systemic inflammatory responses (14). Moreover, Pam_3_CSK_4_ (a TLR2 agonist), monophosphoryl lipid A (a TLR4 agonist), and other TLR agonists have been used as adjuvants for assisting PAc to elicit marked specific immune responses (6, 15). In recent years, nanoparticle (NP)-based adjuvants have been widely explored for assisting antigens in vaccination to treat cancer, bacterial infections, or viral infections due to their specific biological properties. However, few nanoparticle-based adjuvants have been used for caries vaccines.

Zeolitic imidazolate framework-8 (ZIF-8) nanoparticles (NPs) are a subclass of metal-organic framework (MOF) NPs that show excellent pH sensitivity, biodegradability, and various other advantages and have been used in the field of vaccine adjuvants (16, 17). Luzuriaga et al. reported that ZIF-8 protects tobacco mosaic virus from denaturing conditions and improves its immunogenicity to elicit high titers of mouse antibodies, while no observable tissue damage was observed in the skin or vital organs of mice (18). Zhang et al. prepared ZIF-8-based nanoparticles to target dendritic cells (DCs) and induce adequate immune responses as a method to provide a powerful platform against invasive malignancy and rechallenged tumors (19). In addition, ZIF-8 NPs were functionalized with poly(ethylene glycol) (20), aluminum (21), cytosine-phosphate-guanine oligodeoxynucleotides (CpG) (22), or peptides (23) that were utilized to encapsulate several antigens, such as tumor-associated antigens (24), bacterial antigens, or viruses (25), as a vaccine delivery system to enhance antigen-specific immune responses. However, few ZIF-8 NP-based adjuvants have been applied to encapsulate the PAc antigen from S. mutans to induce an immune response and protect against caries.

Here, we prepared an anticaries vaccine based on ZIF-8 NPs in aqueous solution. The ZIF-8 NPs exhibit high stability and dispersibility and a high loading ratio of PAc antigen. The key advantages rely on the pH-sensitive release of the PAc antigen into lysosomes, subsequently accelerating further antigen presentation and improving immune responses *in vitro* and *in vivo*. The obtained anticaries vaccine might result in efficient DC uptake and release in lysosomes and significantly elicit PAc-specific humoral and cellular immunity. Moreover, the anticaries vaccine augments caries protection in rats orally infected with S. mutans. This work provides a promising approach to exploit ZIF-8 NPs as a vaccine adjuvant for providing protection against bacterial infection.

## RESULTS

### Characterization of the ZIF-8@PAc vaccine.

**FIG 1:**
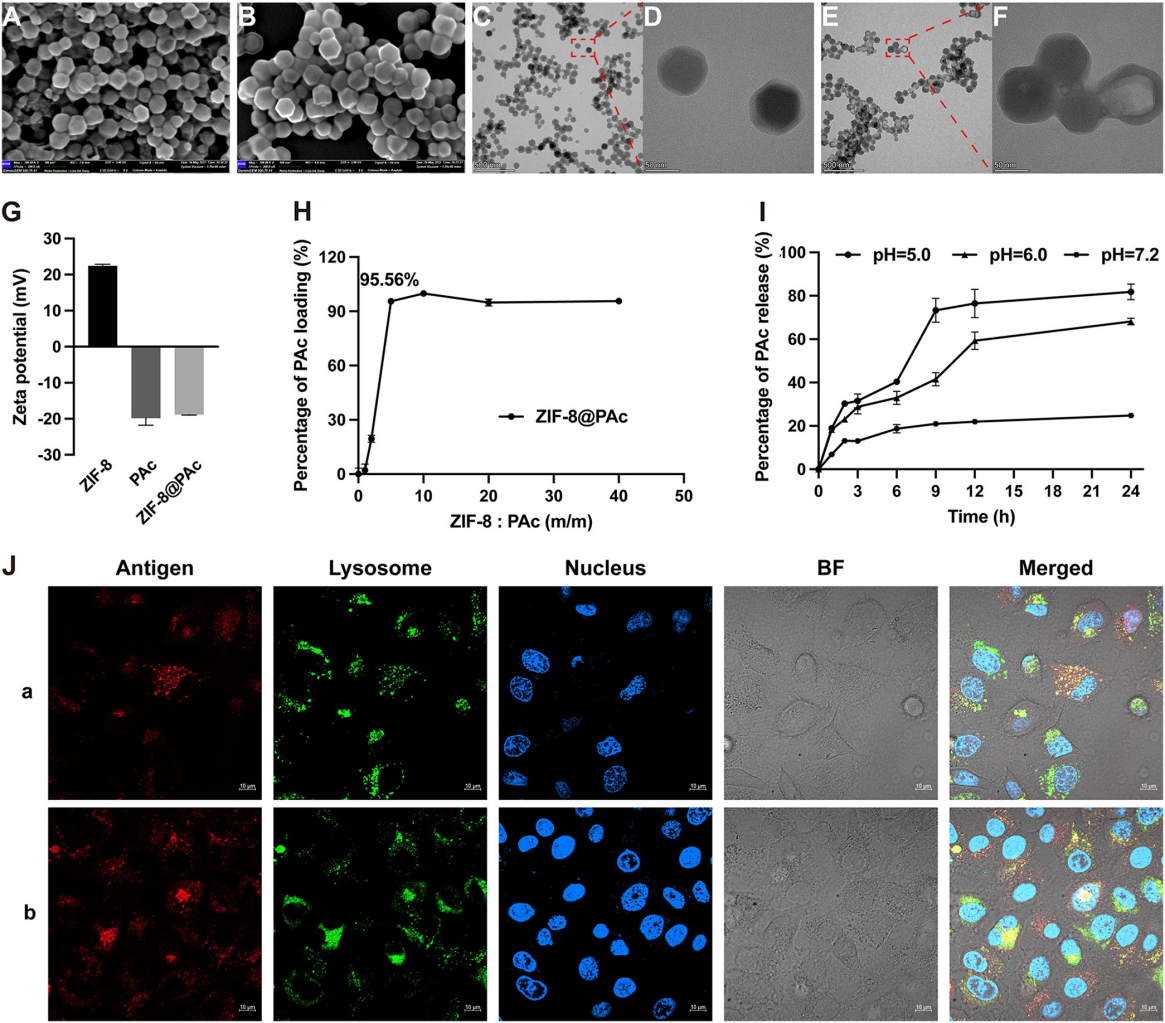
Characterization and intracellular internalization of ZIF-8@PAc. (A and B) SEM images of ZIF-8 NPs (A) and ZIF-8@PAc (B). Bar, 100 nm. (C to F) TEM images of ZIF-8 NPs (C and D) and ZIF-8@PAc (E and F). Bar, 500 or 50 nm. (G) Zeta potentials of ZIF-8 NPs, PAc, and ZIF-8@PAc. (H) PAc loading rate at different weight ratios of ZIF-8 NPs to PAc. (I) *In vitro* release of PAc from ZIF-8@PAc in PBS at different pH values. (J) CLSM images of DC2.4 cells after an incubation with OVA-Cy5 (a) or ZIF-8@OVA-Cy5 (b) to show the intracellular internalization and colocation with lysosomes. OVA was labeled with Cy5 (red). Nuclei and lysosomes were stained with Hoechst 33342 (blue) and LysoTracker (green), respectively. BF, Bright Field. Bars, 10 μm.

ZIF-8 NPs were synthesized in an aqueous solution by mixing Zn^2+^ and 2-methylimidazole as previously described (26). Recombinant PAc was constructed, expressed, and purified according to our previous study (see Fig. S1 in the supplemental material). ZIF-8@PAc was prepared through electrostatic attraction between ZIF-8 NPs with a positive charge and the PAc protein with a negative charge (Fig. 1G). As shown in the scanning electron microscopy (SEM) images (Fig. 1A and B), the obtained ZIF-8@PAc NPs were monodisperse with an average size of approximately 100 nm, similar to that of pure ZIF-8 NPs. The morphology of ZIF-8 NPs and ZIF-8@PAc was further confirmed by performing transmission electron microscopy (TEM) measurements (Fig. 1C, D, E, and F). In addition, the quantity of PAc loaded onto ZIF-8 NPs was measured to optimize the weight ratio of ZIF-8 NPs and PAc for further use *in vitro* and *in vivo*. As shown in Fig. 1H, with increasing ZIF-8 NP concentrations, PAc was gradually loaded onto ZIF-8 NPs and approached saturation when the weight ratio reached 5:1.

ZIF-8@PAc NPs were incubated with phosphate-buffered saline (PBS) at different pH values (pH 7.2, 6.0, or 5.0), and the release of PAc was measured at different time points using the bicinchoninic acid (BCA) assay to verify the antigen release behavior from ZIF-8@PAc NPs. As shown in Fig. 1I, less than 20% of the PAc antigen was released from ZIF-8@PAc NPs at pH 7.2 within 24 h, indicating that the ZIF-8@PAc NPs remained relatively stable under physiological conditions. However, the triggered release of PAc based on the pH-sensitive property of ZIF-8 NPs was measured at pH 6.0 (more than 65%) or pH 5.0 (more than 80%) at 24 h.

The results of the hemolysis assay (Fig. S2) showed that the administration of PAc and ZIF-8 NPs at different concentrations did not cause significant hemolysis, similar to the negative-control PBS, while significant hemolysis was observed in the positive-control group, revealing that the ZIF-8 NPs would not cause hemolysis in mice. In addition, a histopathological assessment was implemented to evaluate the possible damage to tissues and organs caused by the vaccine (27). As shown in Fig. S3, no lesions were observed in the tissues of mice immunized with ZIF-8@PAc, cholera toxin B subunit plus PAc (CTB+PAc), or aluminum adjuvant plus PAc (Alum+PAc) compared to the PBS+PAc control group after hematoxylin and eosin (H&E) staining, which implied that the immunizing dose of ZIF-8@PAc could be administered to the mice without damaging the body.

In addition, vaccine internalization and intracellular trafficking were evaluated. As shown in Fig. 1J, the incubation with ovalbumin (OVA) alone resulted in much lower uptake than that with ZIF-8@OVA, indicating that ZIF-8 NPs enhanced the cellular uptake of the antigen by antigen-presenting cells (APCs). In addition, a larger amount of internalized OVA was released in lysosomes in the ZIF-8@OVA group, which implied that more antigens were processed in lysosomes for further presentation.

### Humoral and cellular immunity.

**FIG 2:**
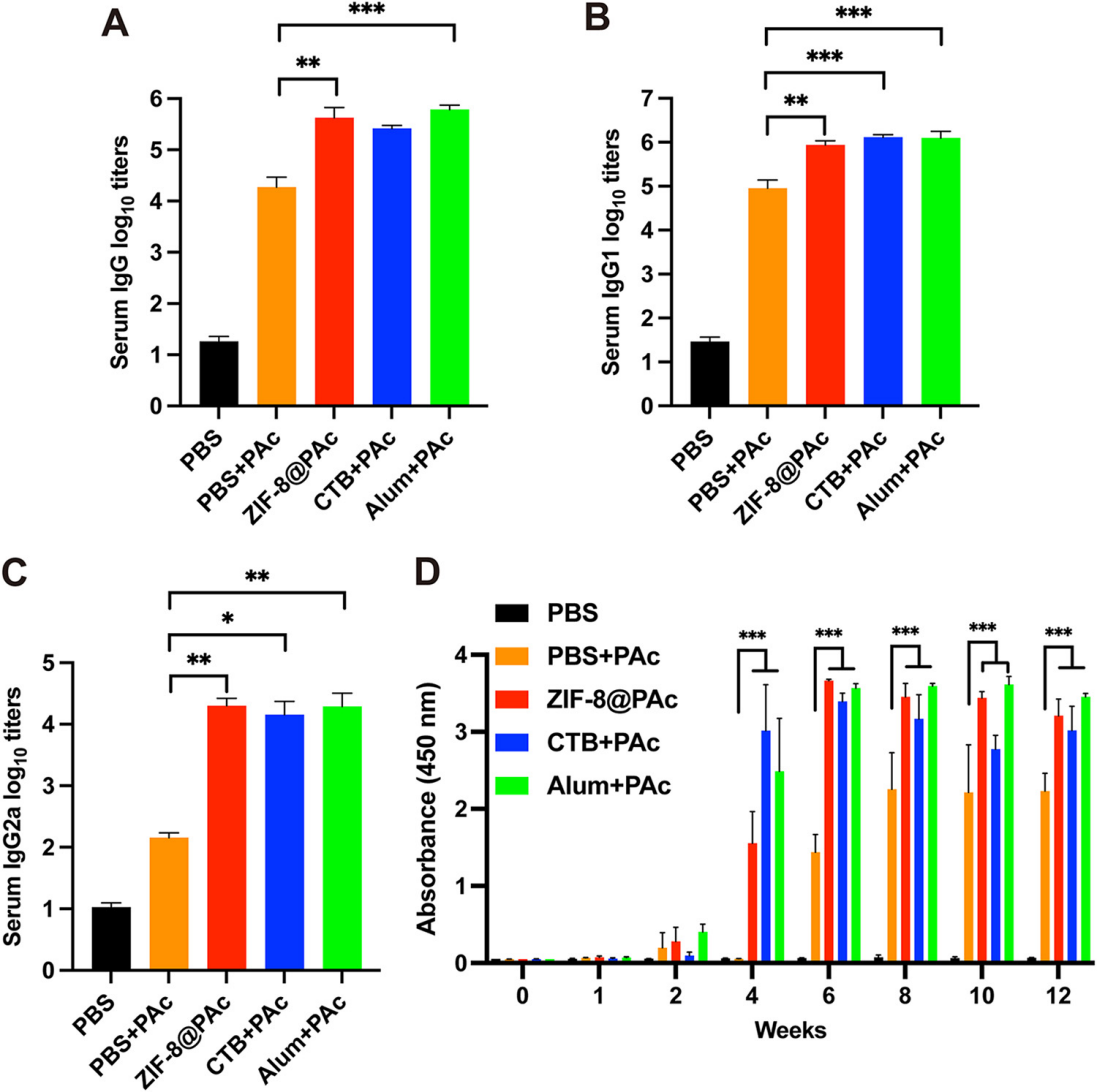
(A to C) PAc-specific antibody titers of IgG, IgG1, and IgG2a. Serum samples were collected from mice immunized with different vaccines, and the antibody titer was measured using an ELISA. (D) PAc-specific antibody levels of IgG at different time points (0, 1, 2, 4, 6, 8, 10, or 12 weeks) were determined using an ELISA (*n* = 5; *, *P* < 0.05; **, *P* < 0.01; ***, *P* < 0.001).

The PAc-specific antibody titer of serum collected from immunized mice was assessed using enzyme-linked immunosorbent assay (ELISA). As shown in Fig. 2A to C, ZIF-8@PAc induced a significantly higher titer of IgG and different subclasses (IgG1 and IgG2a), which was similar to the CTB+PAc or Alum+PAc positive-control group, than in the group immunized with OVA alone. The subclasses of IgG1 and IgG2a are indices used to analyze Th1- or Th2-biased immunity. As shown in Fig. 2B and C, the IgG1 antibody titers were higher than those of IgG2a in each group, indicating that the ZIF-8@PAc vaccine increased Th2-biased immunity. Furthermore, the same level of PAc-specific IgG was observed in mice throughout the vaccination period. As shown in Fig. 2D, the level of PAc-specific IgG was measured at weeks 0, 1, 2, 4, 6, 8, 10, and 12. Very little PAc-specific IgG was produced after the first immunization in the first 2 weeks. A high level of PAc-specific IgG was observed after the second immunization, and this high level was maintained after the third immunization until the 12th week in the present study.

**FIG 3:**
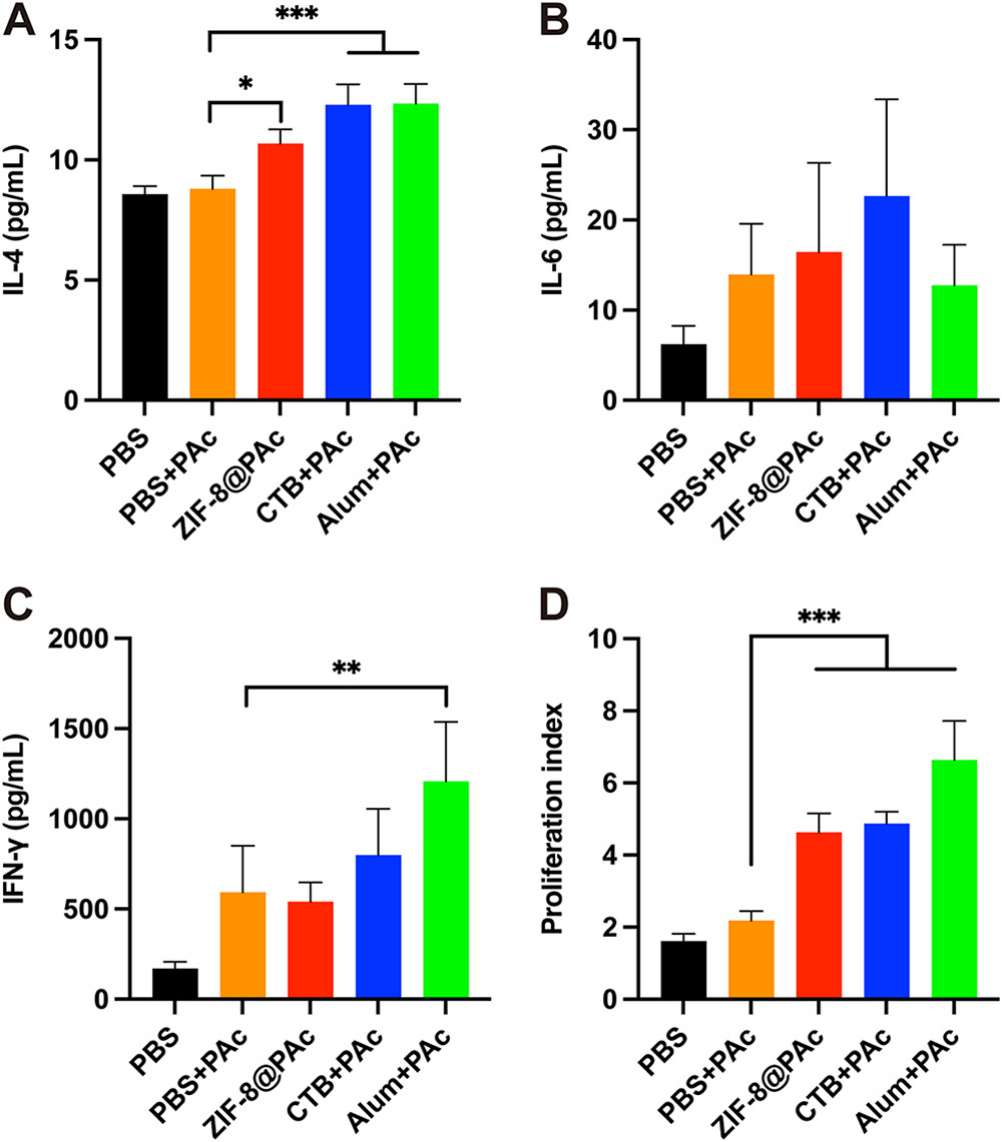
(A to C) Secretion of cytokines IL-4 (A), IL-6 (B), or IFN-γ (C) by splenocytes harvested from immunized mice upon stimulation with PAc. (D) Proliferation index of the splenocytes (*n* = 5; *, *P* < 0.05; **, *P* < 0.01; ***, *P* < 0.001).

The levels of these cytokines secreted from splenocytes were detected using ELISAs. As shown in Fig. 3A to C, the secretion of interleukin-4 (IL-4) from the group treated with ZIF-8@PAc and the positive control was significantly higher than that from the group treated with PAc alone. In addition, the secretion of IL-6 was increased after treatment with different vaccines compared to PAc alone. However, the secretion of the cytokine gamma interferon (IFN-γ) was significantly increased in the Alum+PAc positive-control group, while other treatments showed no obvious increase.

**FIG 4:**
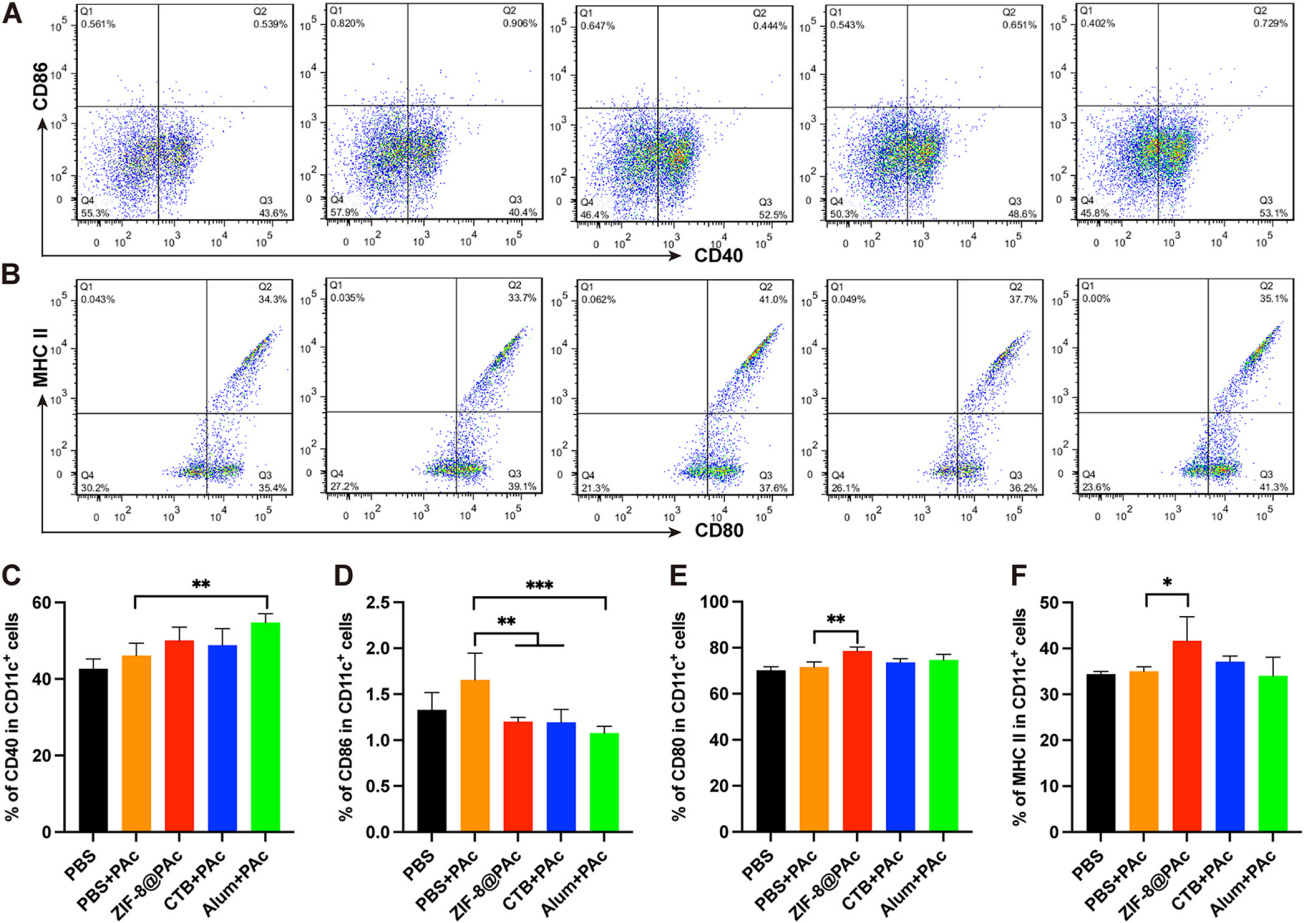
Expression of MHC II molecules and costimulatory molecules (CD40, CD80, and CD86) on DCs in the splenocytes from immunized mice. (A and B) Representative dot plots are shown. (C to F) Statistical analysis is displayed for the dot plots (*n* = 5; *, *P* < 0.05; **, *P* < 0.01; ***, *P* < 0.001).

**FIG 5:**
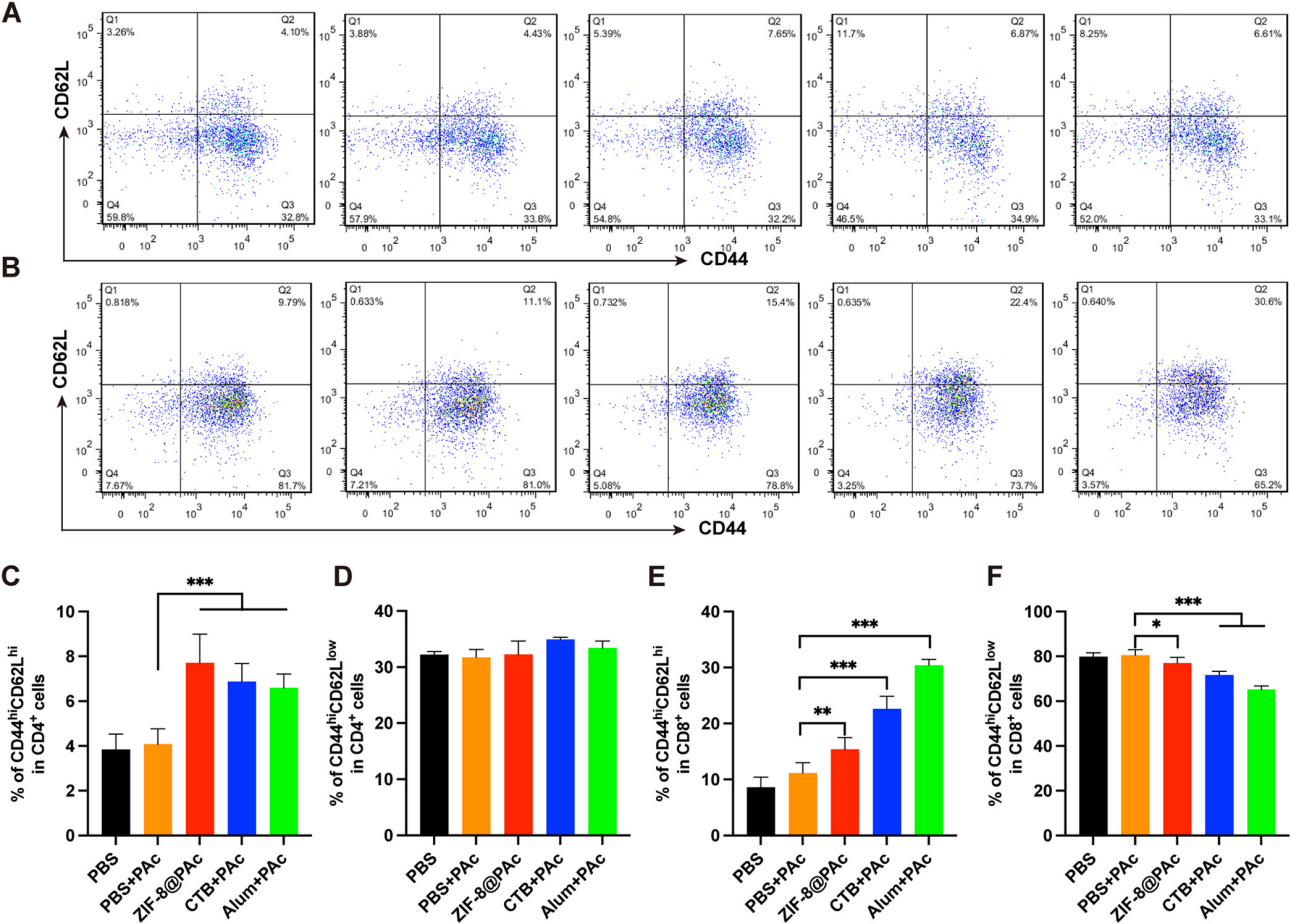
T_CM_ (CD44^hi^ CD62L^hi^) and T_EM_ (CD44^hi^ CD62L^low^) on CD4^+^ or CD8^+^ T cells in splenocytes from immunized mice were detected using flow cytometry. Representative dot plots of CD4^+^ (A) or CD8^+^ (B) T cells and the statistical analysis (C to F) are shown (*n* = 5; *, *P* < 0.05; **, *P* < 0.01; ***, *P* < 0.001).

The PAc-specific proliferation index was measured by a cell counting kit 8 (CCK-8) assay after splenocytes were incubated with PAc antigen. As shown in Fig. 3D, splenocytes significantly proliferated after immunization with ZIF-8@PAc or the positive-control vaccine compared to the group immunized with PAc alone. Moreover, the expression of major histocompatibility complex class II (MHC II), CD40, CD80, and CD86 molecules on CD11c^+^ (a marker of DCs) DCs was detected to evaluate mature DCs in the spleen. Representative dot plots are displayed in Fig. 4A and B, and the statistical analysis is shown in Fig. 4C to F. The expression of the costimulatory molecules CD40 and CD80 was obviously increased in the ZIF-8@PAc or positive-control group, while CD86 expression was significantly decreased. In addition, the expression of MHC II molecules was significantly increased after treatment with ZIF-8@PAc. Furthermore, the levels of central memory T cells (T_CM_) and effective memory T cells (T_EM_) among splenocytes were detected, and representative dot plots and the statistical analysis are shown in Fig. 5. The numbers of the subclasses of CD4^+^ T_CM_ and CD8^+^ T_CM_ in animals immunized with ZIF-8@PAc or the positive control were significantly higher than those in animals immunized with naked PAc, while the number of T_EM_ did not change substantially.

### Anticaries efficacy in rats.

The heart, liver, spleen, lung, and kidney weight indices of rats were calculated according to the weight of organs collected from rats after immunization and infection with S. mutans. As shown in Fig. S4, no obvious difference in the organ weight index was observed after different treatments.

**FIG 6:**
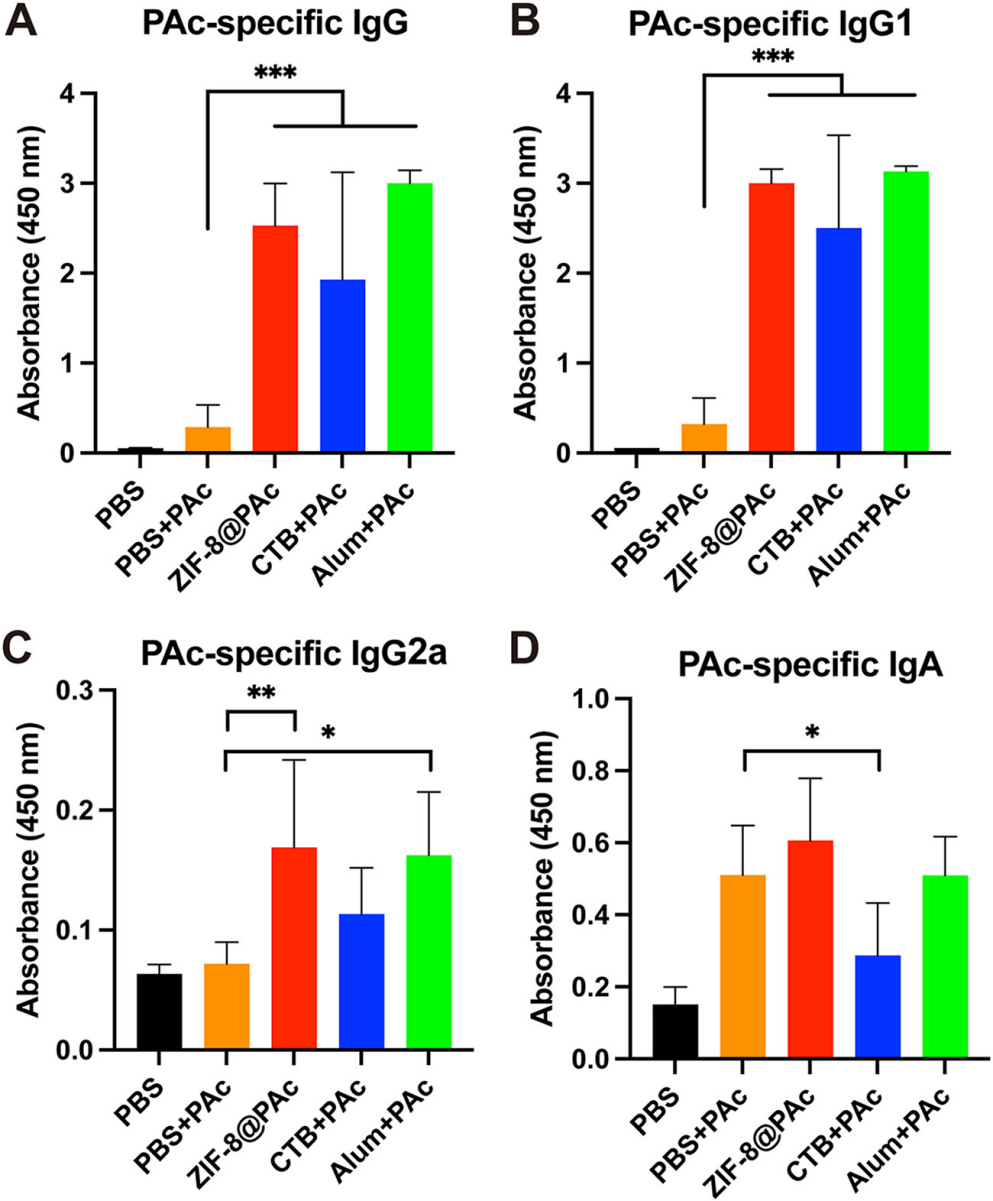
(A to C) PAc-specific antibody levels of IgG, IgG1, or IgG2a in serum samples collected from rats immunized with different vaccines. (D) PAc-specific salivary IgA antibody levels in rats immunized with different vaccines (*n* = 5; *, *P* < 0.05; **, *P* < 0.01; ***, *P* < 0.001).

In addition, serum IgG and salivary IgA antibody titers were measured using ELISAs to evaluate the activation of the immune response. As shown in Fig. 6A to C, the titers of PAc-specific IgG and subclasses (IgG1 and IgG2a) were significantly higher in the ZIF-8@PAc group than in the naked PAc group, which was similar to the positive-control Alum+PAc group and higher than the positive-control CTB+PAc group. Moreover, the PAc-specific salivary IgA titer was higher in the ZIF-8@PAc group than in the other groups, while CTB+PAc induced a low salivary IgA titer.

**FIG 7:**
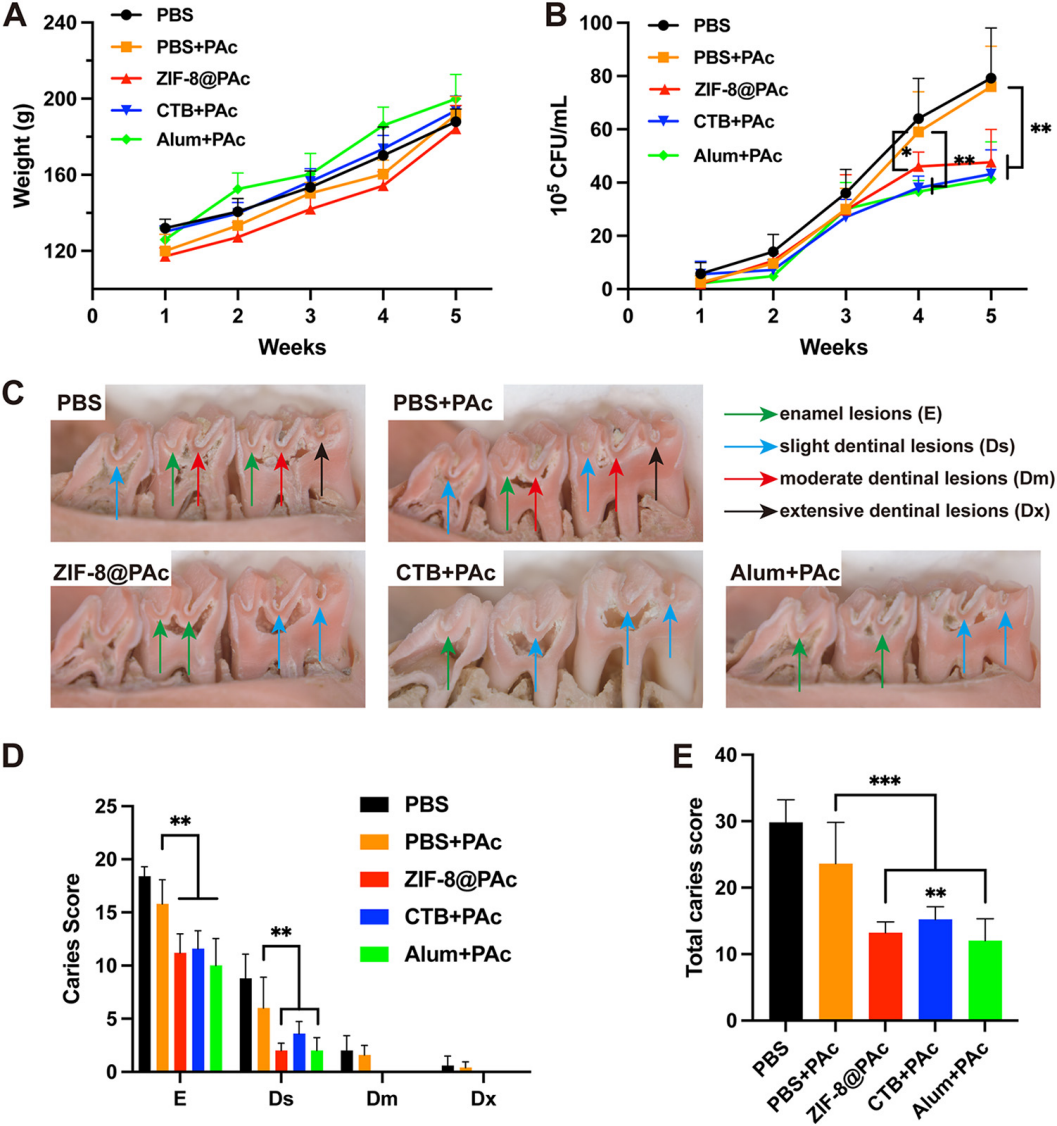
(A) The weight of rats treated with different vaccines was recorded over 5 weeks. (B) Numbers of S. mutans CFU in oral cavities of rats, which were calculated from oral swabs. (C) Representative micrographs of caries lesions on molars from rats in each group. E, Ds, Dm, or Dx is indicated by green, blue, red, or black arrows, respectively. (D) Caries scores of E, Ds, Dm, and Dx for rats immunized with different vaccines. (E) Total caries scores of different groups (*n* = 5; *, *P* < 0.05; **, *P* < 0.01; ***, *P* < 0.001).

The number of S. mutans CFU sustained on the teeth was detected using a colony formation assay, and the results are shown in Fig. 7B. As we inferred, the numbers of S. mutans CFU in the PBS- or naked PAc-immunized rats increased gradually and were maintained at high levels over 5 weeks. The numbers of S. mutans CFU were significantly reduced in the ZIF-8@PAc or positive-control group at the 4th and 5th weeks compared to the naked PAc group. Corresponding to the antibody responses and S. mutans CFU, rats immunized with ZIF-8@PAc, CTB+PAc, or Alum+PAc showed significantly fewer caries lesions, including enamel lesions (E) and slight dentinal lesions (Ds), than the naked PAc group (Fig. 7C and D). No moderate dentinal lesions (Dm) or extensive dentinal lesions (Dx) were observed in rats immunized with ZIF-8@PAc, CTB+PAc, or Alum+PAc (Fig. 7C and D). Accordingly, as shown in Fig. 7E, significantly lower total caries scores (E + Ds + Dm + Dx) were recorded for the rats immunized with ZIF-8@PAc, CTB+PAc, or Alum+PAc than for those immunized with PAc alone.

## DISCUSSION

This study aimed to fabricate an innovate nanoparticle-based anticaries vaccine and investigate systemic immune responses induced by the vaccine. As shown in Fig. 1 and in Fig. S1 in the supplemental material, the ZIF-8 NPs were synthesized and characterized as spherical nanoparticles, PAc antigen was expressed successfully, and then the vaccine ZIF-8@PAc was prepared by electrostatic attraction between ZIF-8 NPs with a positive charge and PAc with a negative charge. In addition, PAc was nearly completely loaded onto ZIF-8 NPs when the weight ratio of ZIF-8 NPs to PAc was greater than 5:1, which was subsequently used in this study. Furthermore, the pH-triggered release of the loaded PAc antigen was important for antigen processing, antigen presentation, and antigen-specific immune responses. ZIF-8 NPs are pH-responsive nanoparticles that can be degraded at pH values below 6.0. In this study, the release of PAc from ZIF-8@PAc was measured at different pH values (pH 7.2, 6.0, or 5.0). The results suggested that ZIF-8@PAc NPs were stable under physiological conditions and showed pH-sensitive antigen release behavior that will be beneficial for intracellular processing and subsequent immune responses.

The biocompatibility of ZIF-8 NPs was monitored by performing a hemolysis assay and H&E staining before cell and animal experiments. These results from the hemolysis and H&E assays suggested that the ZIF-8@PAc NPs have good biocompatibility and great potential as a novel vaccine. In addition, vaccine internalization and intracellular trafficking by antigen-presenting cells (APCs), specifically dendritic cells (DCs), are critical for immune activation (20). We incubated DC2.4 cells with ovalbumin labeled with Cy5 (OVA-Cy5, as a model antigen) or ZIF-8@OVA-Cy5 for 6 h to evaluate the uptake and trafficking of ZIF-8@PAc by APCs. The result indicated that ZIF-8 NPs enhanced antigen uptake into APCs and pH-sensitive release into lysosomes for further processing and presentation to activate immune responses.

After preparation and characterization of ZIF-8@PAc vaccine *in vitro*, the immunological response to the ZIF-8@PAc vaccine was evaluated in mice after subcutaneous injection. ELISA was used to measure the PAc-specific antibody levels in the serum collected from vaccinated mice. The results showed that the ZIF-8@PAc vaccine led to significantly higher levels of IgG and its subclasses and resulted in a Th2-biased immune response that was maintained at high levels throughout the vaccination period. In addition, the level of serum IgG titer was also higher than that using TLR agonists as an adjuvant (12, 14). Based on this result, ZIF-8@PAc not only elicited a high level of PAc-specific IgG but also maintained this high level for immune protection. Additionally, the activation of the immune system during vaccination is often accompanied by cytokine secretion. The levels of IL-4 and IL-6, typical regulators of the Th2 response, and IFN-γ, a regulator of the Th1 response, were measured using ELISA. The results revealed that ZIF-8@PAc induced the secretion of IL-4 and IL-6, while the secretion of IFN-γ was similar to that seen in the group receiving only PAc. The results indicated that the ZIF-8@PAc vaccine elicited a Th2-biased immune response, also confirmed by the high IgG1 antibody titer, and provided long-lasting immune protection.

In addition, proliferation and clustering of splenocytes are the main factors that elicit high levels of cytokine secretion and memory cell production. The proliferation index and numbers of mature DCs and memory T cells among splenocytes collected from vaccinated mice were determined by performing a CCK-8 assay or flow cytometry. The proliferation of splenocytes indirectly reflects the extent of the antigen-specific immune response when the cells are exposed to the same antigen again. In the present study, the proliferation of splenocytes indicated that splenocytes from vaccinated mice proliferated in a PAc-specific manner to protect against PAc reexposure, which was confirmed by the results of subsequent anticaries protection assays. Moreover, DCs are key antigen-presenting cells that mature after capturing antigens, expressing MHC II molecules and costimulatory molecules such as CD40, CD80, and CD86, for further antigen presentation to T lymphocytes (28). The higher numbers of mature DCs in the spleen observed in this study indicated that more antigens were captured, leading to a stronger immune response. MHC II molecules mainly participate in the processing and presentation of exogenous antigens, which induce high levels of humoral immunity. ZIF-8@PAc also led to higher expression of MHC II and costimulatory molecules, promoting MHC II-type antigen presentation and a stronger humoral immune response. Furthermore, memory T cells play a critical role in adaptive immune memory and provide rapid and powerful protection against reexposure to antigens. Central memory T cells (T_CM_) with the phenotype CD44^hi^ CD62L^hi^ that are located in secondary lymphoid organs differentiate into effector T cells after reexposure to antigens to mediate reactive memory, while effective memory T cells (T_EM_) with the phenotype CD44^hi^ CD62L^low^ trigger immediate protective memory against the second infection by inducing cytokine production (29, 30). The significantly higher numbers of CD4^+^ T_CM_ and CD8^+^ T_CM_ in animals immunized with ZIF-8@PAc suggested that ZIF-8@PAc elicited a greater number of T_CM_ in the spleen, enabling a more effective response to reexposed PAc.

Finally, to assess the anticaries efficacy of the ZIF-8@PAc vaccine, rats were immunized with PBS, PBS+PAc, ZIF-8@PAc, CTB+PAc, or Alum+PAc. After the immunization, the rats were challenged with S. mutans on teeth to evaluate their protection against dental caries. Then, the weight of the rats was recorded, saliva was collected for the detection of bacterial colonies and PAc-specific IgA, serum was collected to measure PAc-specific IgG antibody titers, and the degree of caries was evaluated through dental images of the rats. Our results indicated that there were no indications of systemic toxicity resulting from immunization or infection with S. mutans, as verified by the stable weight of rats receiving different vaccines. Furthermore, the ZIF-8@PAc vaccine elicited a much stronger immune response, as evidenced by the significantly higher titers of PAc-specific IgG, IgG subclasses, and salivary IgA. These results suggested that the ZIF-8@PAc vaccine may effectively protect against caries by eliciting a robust immune response in rats. Finally, the efficacy of the anticaries potential of ZIF-8@PAc was determined by measuring the number of S. mutans colonies and evaluating the extent of dental caries. The numbers of S. mutans CFU were significantly reduced in the ZIF-8@PAc group, and significantly lower total caries scores were recorded in the ZIF-8@PAc group. The results for the antibody titer, S. mutans CFU, and caries degree indicated that the ZIF-8@PAc vaccine had the potential to provide robust protection against caries, while PAc alone did not induce significant protection against caries.

### Conclusions.

In summary, the use of ZIF-8 NPs with good biocompatibility, pH responsiveness, and high loading of the PAc antigen as an adjuvant markedly promoted PAc intracellular internalization and presentation and elicited strong immune responses, including high levels of antibody titers and cytokines, a high splenocyte proliferation index, and high levels of mature DCs and T_CM_. Furthermore, the PAc-specific immune response effectively inhibited the colonization by S. mutans on teeth and provided considerable protection against caries in rats. These advantages suggest that ZIF-8@PAc is a promising anticaries vaccine candidate.

## MATERIALS AND METHODS

### Materials.

Ampicillin, chloramphenicol, carbenicillin, streptomycin, and TrueColor prestained protein marker were purchased from Sangong Bioengineering (Shanghai, China). Zinc nitrate hexahydrate [Zn(NO_3_)_2_·6H_2_O], 2-methylimidazole, methanol, dl-arabinose, imidazole, and murexide were purchased from Aladdin Biochemical Technology (Shanghai, China). A nickel-nitrilotriacetic acid (Ni-NTA) affinity chromatography purification column was purchased from TransGen Biotech (Beijing, China). Enterokinase, an SDS-PAGE protein kit, tetramethylbenzidine (TMB) two-component substrate solution, ELISA coating buffer, and ELISA stop solution were purchased from Solarbio (Beijing, China). Paraformaldehyde (4%) was purchased from Asegene (Guangzhou, China). An enhanced BCA protein assay kit, red blood cell lysis buffer, and recombinant cholera toxin B subunit (rCTB) were purchased from Beyotime Biotechnology (Shanghai, China). Aluminum adjuvant was purchased from InvivoGen (Toulouse, France). Horseradish peroxidase (HRP)-conjugated goat anti-mouse IgG, IgG1, and IgG2a antibodies were purchased from Thermo (MA, USA). Fluorescent dye-labeled CD11c, MHC II, CD3, CD4, CD8, CD44, and CD62L antibodies were purchased from BioLegend (CA, USA). IL-4, IL-6, and IFN-γ enzyme-linked immunosorbent assay (ELISA) kits were purchased from BioLegend (CA, USA). The cell counting kit 8 (CCK-8) was purchased from Dojindo (Japan). OVA-Cy5 was purchased from QiyueBio (Xi’an, China).

DC2.4 cells were cultured in RPMI 1640 (Gibco, CA, USA) supplemented with 10% fetal bovine serum (FBS) and a 1% penicillin-streptomycin solution (Gibco, CA, USA) at 37°C with 5% CO_2_. LysoTracker and Hoechst 33342 were purchased from Thermo (MA, USA).

### Characterization.

The surface morphologies of ZIF-8 and ZIF-8@PAc were observed using a scanning electron microscope (SEM) (Zeiss, Germany). Transmission electron microscopy (TEM) images were recorded using an FEI Talos F200X transmission electron microscope. The zeta potentials of PAc, ZIF-8, and ZIF-8@PAc were recorded using a Zetasizer Nano ZS (Malvern, UK). The UV-visible (UV-Vis) absorption spectra were measured with a UV-3100 spectrophotometer (Mapada, China). Flow cytometry was performed using a BD FACSAria III flow cytometer (NJ, USA).

### Loading and release of PAc.

ZIF-8@PAc with different mass ratios of ZIF-8 NPs and PAc (0:1, 0.5:1, 1:1, 2:1, 5:1, 10:1, 20:1, and 40:1 [*n* = 3]) was prepared and incubated for 20 min at room temperature to evaluate the loading rate. After centrifugation (8,000 rpm, 5 min), the supernatant was collected, the protein concentration in the supernatant was measured using an enhanced BCA protein assay kit, and the loading rate was calculated to obtain the optimal loading condition. In addition, the release of PAc from ZIF-8@PAc with the optimized loading conditions at different pH values (pH 7.2, pH 6.0, or pH 5.0) was evaluated at different time points (1 h, 2 h, 3 h, 6 h, 9 h, 12 h, and 24 h). After stirring and centrifugation, the supernatant was collected, and the protein concentration in the supernatant was measured to calculate the release curve (*n* = 3).

### Hemolysis assay.

The hemolytic property of ZIF-8 NPs was tested using mouse red blood cells. First, the red blood cell suspension was diluted with PBS to a concentration of 2%. Then, 0.5 mL of a 2% red blood cell suspension was centrifuged, resuspended in PBS (negative control), red blood cell lysis buffer (positive control), PAc (100 μg/mL in PBS), or different concentrations of ZIF-8 NPs (10, 50, 100, 200, 500, or 1,000 μg/mL in PBS), and incubated at 37°C for 30 min. After centrifugation (1,200 rpm, 1 min), photographs were captured, and 100 μL of the supernatant was collected into a 96-well plate to scan the spectrum using a full-band microplate reader (Thermo, USA).

### Cellular uptake.

Confocal laser scanning microscopy (CLSM) confirmed that the nanoparticles promoted antigen internalization. First, DC2.4 cells were seeded into a 24-well plate at a density of 2 × 10^5^ cells/well and cultured for 24 h. Cells were then incubated with OVA-Cy5 or ZIF-8@OVA-Cy5 for 6 h. After three washes with PBS, the cells were incubated with LysoTracker at 37°C for 1 h to label lysosomes. After three additional washes with PBS, the cells were fixed with 4% paraformaldehyde, and the nuclei were labeled with Hoechst 33342. Finally, images of the cells were captured using a confocal laser scanning microscope.

### Mouse immunity.

Female BALB/c mice aged 6 weeks were purchased from SPF (Beijing) Biotechnology Co., Ltd., China. All animal experiments were conducted in accordance with the *Guide for the Care and Use of Laboratory Animals* (31) and were approved by the Beijing Municipal Ethical Committee for Laboratory Animals. Animal experiments were performed at the Zhuhai Campus of Zunyi Medical University. Each mouse was immunized with 20 μg of PAc protein in a total volume of 100 μL by subcutaneous multipoint immunization. PBS, PBS+PAc, ZIF-8@PAc, CTB+PAc (CTB = 5 μg/mouse), and Alum+PAc (Alum = 100 μg/mouse) were administered. Mice (*n* = 5) were immunized on day 0, day 14, and day 28. Serum was collected from the mice after the injection of anesthesia on the 35th day, and the titer of PAc-specific antibodies in the serum was detected using an ELISA. The spleens of the mice were collected for subsequent experiments. In addition, mice were immunized with the same method, blood samples were collected from mouse tails at 0, 1, 2, 4, 6, 8, 10, and 12 weeks, and the titer of IgG antibodies was detected.

### Antibody titer.

First, a 10-μg/mL PAc protein solution was prepared with 0.1 M ELISA coating buffer, which was placed in each well of a 96-well ELISA plate overnight at 4°C. After removing the coating solution, the plate was washed once with PBST (PBS containing 0.5 mL/L Tween 20) and blocked with PBST containing 5% milk for 1 h at room temperature. After four washes with PBST, 100 μL of a serum sample was added to each well, and the plate was incubated at 37°C for 2 h. Then, the plate was washed with PBST four times, and 100 μL of antibody (IgG [1:5,000], IgG1 [1:1,000], or IgG2a [1:1,000]) was added. The plate was incubated at 37°C for 1 h. After five washes with PBST, 100 μL of TMB was added and incubated for 15 min, followed by the addition of 100 μL of ELISA stop solution, and the absorbance of each well at 450 nm was detected using a microplate reader.

### H&E staining.

The heart, liver, spleen, lungs, and kidneys of mice were collected after anesthesia, fixed with 4% paraformaldehyde, dehydrated, embedded in paraffin, and stained with hematoxylin and eosin. Finally, the morphological structure of the organs was studied by observing the images under a microscope.

### Splenocyte proliferation.

On the 35th day, the mice were anesthetized, and the spleens of the mice were harvested to collect splenocytes. The splenocytes were suspended in complete RPMI 1640 medium and diluted to 1 × 10^7^ cells/mL for further use. Splenocytes (100 μL) were added to each well of a 96-well plate, and 100 μL of PAc protein (20 μg/mL) was added for stimulation. After an incubation at 37°C with 5% CO_2_ for 72 h, 20 μL of CCK-8 solution was added to each well, and the plates were incubated for an additional 2 h. The absorbance was measured at 450 nm with a microplate reader to calculate the splenocyte proliferation index.

### Cytokines.

The splenocytes obtained as described above were seeded into a 12-well plate at a density of 5 × 10^6^ cells/mL. PAc antigen (25 μg/mL) was added, and the plate was incubated for 60 h. Then, the cell supernatant was collected. Subsequently, the levels of the cytokines IL-4, IL-6, and IFN-γ in the supernatant were measured using cytokine ELISA kits.

### Expression of MHC II and costimulatory molecules on DCs.

First, a suspension of 1 × 10^7^ splenocytes/mL was diluted to 1 × 10^6^ cells per tube with PBS and washed once with PBS. Then, the cells were incubated with fluorescent dye-conjugated antibodies (fluorescein isothiocyanate [FITC]–anti-CD11c, phycoerythrin [PE]/Cy5–anti-MHC II, PE–anti-CD80, PE/Cy5–anti-CD86, and PE–anti-CD40) at 4°C for 30 min. After removing unbound antibodies and washing with PBS, the cells were resuspended in 500 μL of PBS for flow cytometry detection.

### Memory T cells.

A suspension of 1 × 10^7^ splenocytes/mL was diluted to 1 × 10^6^ cells/mL. After washes with PBS, the cells were incubated with a 1:200 dilution of different fluorophore-labeled antibodies, namely, allophycocyanin (APC)–anti-CD3, FITC–anti-CD4, peridinin chlorophyll protein (PerCP)-Cy5.5–anti-CD8α, PE–anti-CD44, or APC–anti-CD62L, at 4°C for 30 min. After removing unbound antibodies and washing with PBS, the cells were resuspended in 500 μL of PBS for flow cytometry detection.

### Anticaries effect in rats.

Eighteen-day-old female Sprague-Dawley rats were purchased from Zhuhai BesTest Bio-Tech Co., Ltd., China. All animal experiments were conducted in accordance with the *Guide for the Care and Use of Laboratory Animals* (31) and were approved by the Ethics Committee of Guangdong Province for Laboratory Animals. Animals were fed using a Keyes diet 2000 caries diet without antibiotics (32), and experiments were performed at the Zhuhai Campus of Zunyi Medical University. On the 20th day, chloramphenicol, ampicillin, and carbenicillin (1 g/kg of body weight) were added to the caries diet, and 200 μg/mL penicillin and 1,500 μg/mL streptomycin were added to the drinking water (antibiotic dosage was 1 g/kg) for 3 days. On the 23rd day, the rats were randomly divided into five groups (PBS, PBS+PAc, ZIF-8@PAc, CTB+PAc, and Alum+PAc [*n* = 5]). Each rat was immunized with 50 μg of PAc by subcutaneous multipoint immunization three times at an interval of 14 days. The rats were infected with S. mutans UA159 (1 × 10^9^ CFU/mL, 3 times a day, 200 μL each time, 30-min interval) from 24 to 26 days. The weight of the rats was recorded, and saliva was collected. Fourteen days after the third immunization, saliva and serum were collected from the rats under anesthesia. The heart, liver, spleen, lung, and kidney were harvested and weighed to calculate organ indices. The mandibles were separated, and the enamel and dentin were scored and counted. The upper jaws of rats were soaked and stained with 0.4% murexide for 12 h, and micrographs of caries in the mesiodistal sagittal plane were captured using a microscope (Zeiss, Germany). Caries was classified as grade E, Ds, Dm, and Dx and scored according to the Keyes classical score (33).

### Statistical analysis.

Statistical analyses were performed using one-way analysis of variance (ANOVA), followed by the Dunnett test using GraphPad Prism 9 software, and the data are presented as the means ± standard deviations (SD). Significance was indicated as follows: *, *P* < 0.05; **, *P* < 0.01; and ***, *P* < 0.001.
