# Supplementary material for: A Nanoparticle-Based Anticaries Vaccine Enhances the Persistent Immune Response To Inhibit Streptococcus mutans and Prevent Caries
Source: Microbiol Spectr. 2023 Mar 28;11(2):e04328-22. doi: 10.1128/spectrum.04328-22 (PMC10100722; doi:10.1128/spectrum.04328-22)
Supplement: Supplemental file 1 — Supplemental material. Download spectrum.04328-22-s0001.pdf, PDF file, 0.7 MB [file spectrum.04328-22-s0001.pdf]

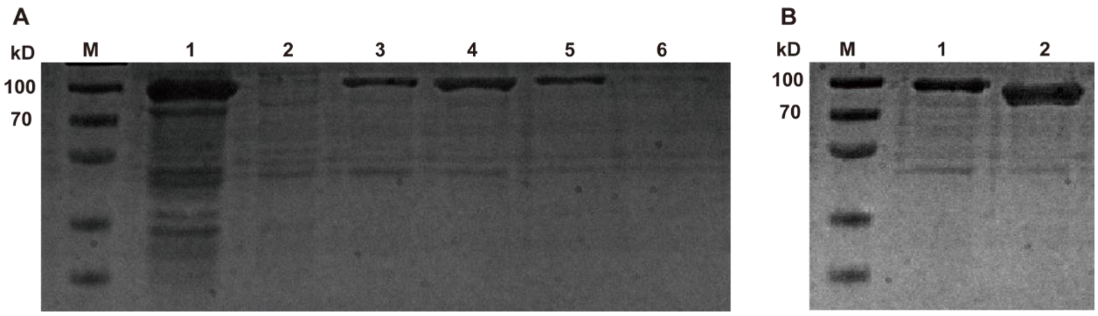

Figure S1. The constructure of plasmid of pBAD/HisA-*pac* and expression of PAc. (A) Purification of PAc-His (elution with different concentration of imidazole (10, 25, 50, 100, 500 mM)). (B) Purification of PAc-His and PAc).

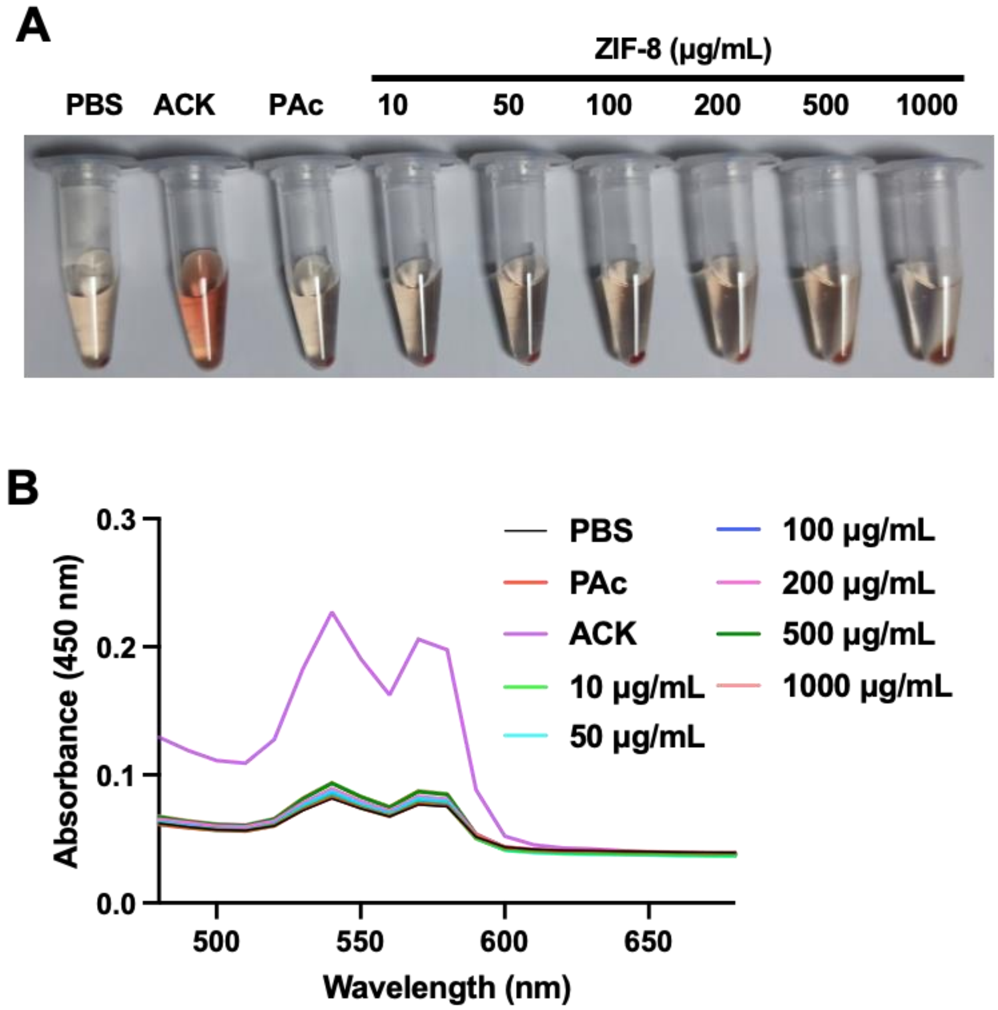

Figure S2. (A) Photos of the hemolysis assay. (B) The absorption of peak of hemoglobin

in the supernatant of RBCs treated with PBS, PAc, ACK (blood cell lysis), ZIF-8 NPs with different concentration.

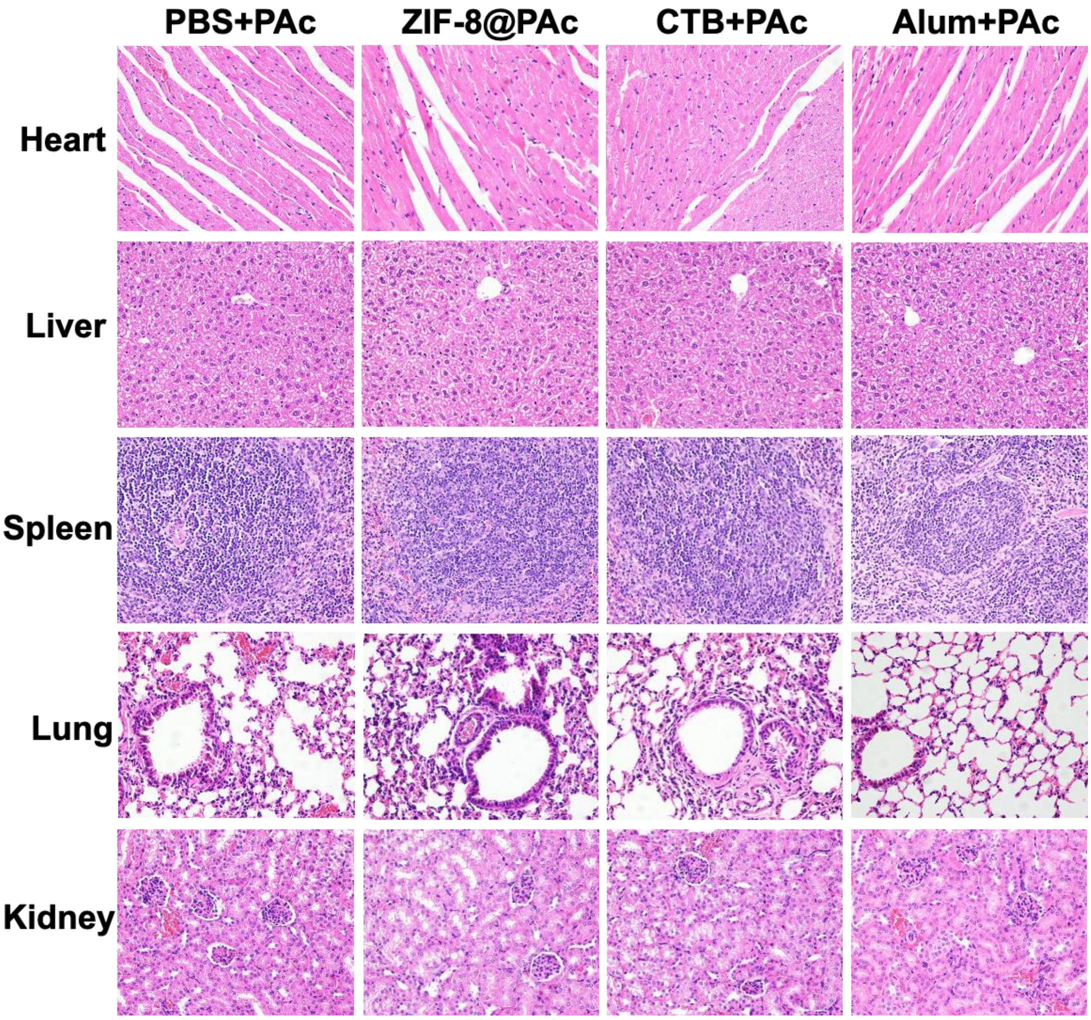

Figure S3. Histopathological images of organs (heart, liver, spleen, lung, and kidney) harvested from immunized mice with different vaccines.

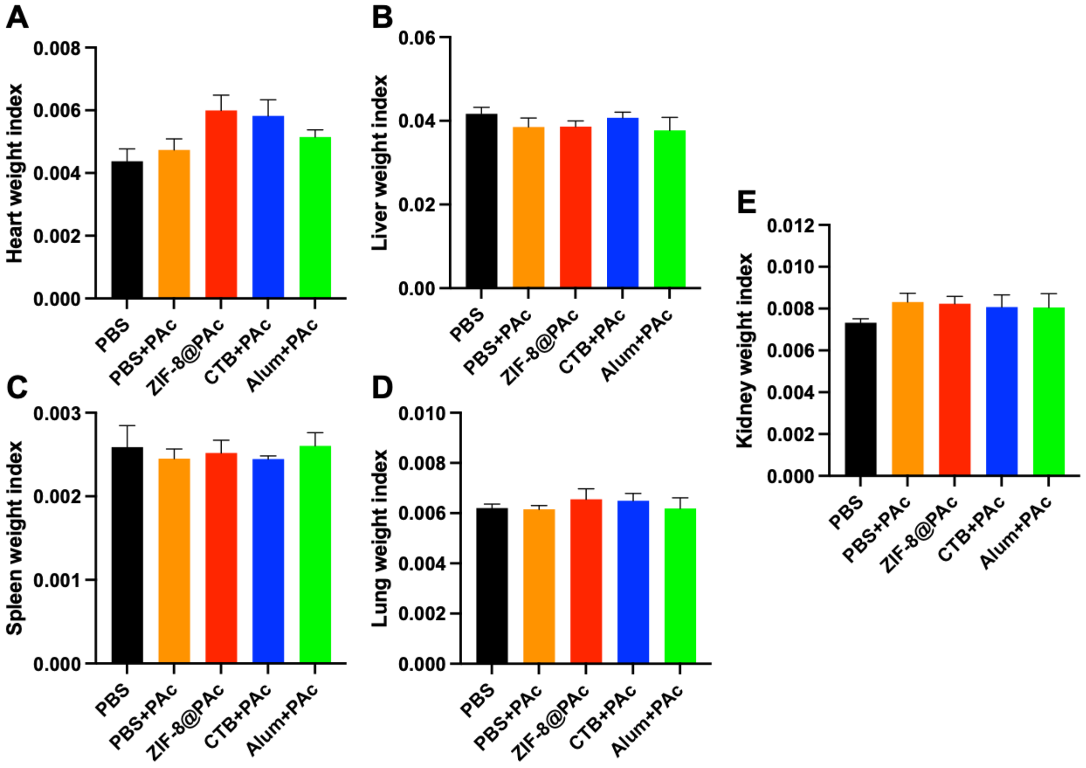

Figure S4. Weight index of organs (heart, liver, spleen, lung, and kidney) harvested from immunized rats with different vaccines.
